# Mobile, Cloud, and Big Data Computing: Contributions, Challenges, and New Directions in Telecardiology

**DOI:** 10.3390/ijerph10116131

**Published:** 2013-11-13

**Authors:** Jui-Chien Hsieh, Ai-Hsien Li, Chung-Chi Yang

**Affiliations:** 1 Department of Information Management, Yuan Ze University, 135 Yuan-Tung Road, Chungli 32003, Taiwan; 2 Cardiovascular Center, Far Eastern Memorial Hospital, Banchao, Taipei 220, Taiwan; E-Mail: las1012.tw@gmail.com; 3 Division of Cardiology, Department of Medicine, Taoyuan Armed Forces General Hospital, Longtan 325, Taiwan; E-Mail: t220979@ms16.hinet.net

**Keywords:** mobile computing, cloud computing, telecardiology, electrocardiograph, echocardiography, medical images, big data

## Abstract

Many studies have indicated that computing technology can enable off-site cardiologists to read patients’ electrocardiograph (ECG), echocardiography (ECHO), and relevant images via smart phones during pre-hospital, in-hospital, and post-hospital teleconsultation, which not only identifies emergency cases in need of immediate treatment, but also prevents the unnecessary re-hospitalizations. Meanwhile, several studies have combined cloud computing and mobile computing to facilitate better storage, delivery, retrieval, and management of medical files for telecardiology. In the future, the aggregated ECG and images from hospitals worldwide will become big data, which should be used to develop an e-consultation program helping on-site practitioners deliver appropriate treatment. With information technology, real-time tele-consultation and tele-diagnosis of ECG and images can be practiced via an e-platform for clinical, research, and educational purposes. While being devoted to promote the application of information technology onto telecardiology, we need to resolve several issues: (1) data confidentiality in the cloud, (2) data interoperability among hospitals, and (3) network latency and accessibility. If these challenges are overcome, tele-consultation will be ubiquitous, easy to perform, inexpensive, and beneficial. Most importantly, these services will increase global collaboration and advance clinical practice, education, and scientific research in cardiology.

## 1. The Development of Telecardiology

Telecardiology refers to the monitoring or diagnosis of patients’ cardiac activities at a distance via telecommunication technology. Waveform-based electrocardiography (ECG) and imaging-based echocardiography (ECHO) are frequently applied tools in cardiology [1]. The ECG, especially 12-lead ECG, which provides clinicians with richer information about electro-cardiac activities as compared to 3-lead ECG, is the most demanded screening tool to detect life-threatening coronary-related diseases [2]. ECHO is also a widely applied tool in telecardiology because it physically evaluates cardiac and vascular anatomical structures and physiological functions, which affect intervention strategies [3]. The greatest advantage of telecardiology is that it enables remote cardiologists to conduct timely diagnosis and propose effective therapeutic strategies for patients in rural areas where professional cardiologists are rarely accessible. In addition to lowering the mortality rate of patients with heart attack, telecardiology can reduce the costly transportation from home to hospital or unnecessary transfers between hospitals [4,5,6]. As a critical tool in telecardiology, wireless telecommunication has delivered pervasive services with less interruption errors as compared to the traditional telephone line. In this review paper, the authors have selected papers specifically on mobile or cloud computing in 12-lead ECG and ECHO telecardiology that represent the current technological development in telecardiology. Based on the review of these papers, the authors will discuss the limitations of current technological development and the directions of future research and clinical practice. The authors start the discussion with the following three questions, which indicate the areas for improvement of telecardiology:

1. How to improve the current ECG and ECHO instrumentation and the software so that any experienced cardiologist can remotely access to the files and offer timely assessment and treatment recommendation when he or she is away from the patient?
2. How to make telecardiology services interoperable across hospitals?
3. How to set up a cost-effective platform that incorporates various functions including clinical services, large-scale clinical research databank, and educational services [7,8]?

In the past ten years, revolutionary progress in telecardiology has been made with the use of information technology, including mobile computing and cloud computing. Mobile devices, such as smart phones and tablets, will continuous to be the essential tools to deliver telecardiology services over wireless networks. The deployment of cloud computing will inexpensively facilitate the collaborative application of telecardiology across hospitals and expand services from regional to global. As a result, people residing in rural districts or under-resourced areas around the world will be able to benefit from telecardiology services.

In this paper, we will review and discuss the development of telecardiology services from the past to the future, with the focus on the use of ECG, ECHO, and relevant medical images. 

## 2. ECG Telemedicine

Traditionally, the clinical applications of ECG telemedicine can be categorized as post-hospital (home-monitoring), in-hospital, and pre-hospital services. With the advances in telecommunication, ubiquitous ECG monitoring is now feasible.

### 2.1. The Development of ECG Tele-Monitoring

**Figure 1:**
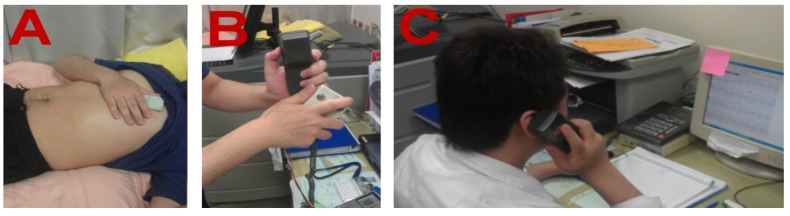
The demonstration of ECG transmission via telephone line.

To enable home ECG monitoring in the environment without internet or with only traditional telephone lines, several researchers have developed a technology to record ECG signals as audio input and then this recorded ECG audio is transmitted to a hospital via a fixed telephone line or a mobile phone [9,10]. Far Eastern Memorial Hospital in Taiwan also adopted this technology to realize home ECG monitoring. As shown in panel A of Figure 1, a card-like ECG device that allows post-hospitalization patients with heart diseases to record single-lead ECGs as an audio files stored on this card, is used by Far Eastern Memorial Hospital in Taiwan. As shown in panel B of Figure 1, the patient transmits the ECG signal by placing the card close to a telephone. Panel C of Figure 1 shows a remote receiving station where an on-duty cardiologist obtains the ECG signals and interprets the files. The reliability of ECG signal transmission depends on the quality of the recorded audio and the correct placement of the ECG card near a phone. While the advantage of this technology is the successful application of telecardiology services without internet, its drawbacks include the possible distortion of the audio, as well as the laborious manual operations by patients, who may have difficulty recording and transmitting audio files repetitively. 

With the emergence of the third generation (3G) wireless telecommunication technology, patients can use mobile phones to transmit data to the hospital via wireless networks and the internet. First, ECG data are captured by an ECG device at home. The data are subsequently delivered to a mobile phone via Bluetooth or ZigBee [11,12,13]. The 3G based mobile phone then transmits ECG data to a remote caregiver’s mobile phone or computer via internet using the Transmission Control Protocol/Internet Protocol (TCP/IP), which guarantees the data will be delivered and received without errors. As compared with the ECG transmission through the traditional telephone line, this TCP/IP-based ECG transmission has higher reliability with less interruption [13].

### 2.2. Clinical Evaluations of 12-Lead ECG Home Monitoring

To date, several clinical trials of 12-lead ECG home monitoring have proved the feasibility, benefits, and reliability of the telecardiology service described previously. For example, 12-lead ECG home monitoring was used to evaluate and assist patients with chest pains [14]. In this study, the 12-lead ECGs of at-home patients who called for emergency medical service were recorded by an ECG device. The ECG files were then transmitted via mobile phone to a cardiologist hub where several senior cardiologists can review the data, make the correct diagnosis, and intervene appropriately. The findings of this study proved the effectiveness of this telecardiology service provided by remote cardiologists. This service not only decreased diagnostic errors of ST elevation myocardial infarction but also reduced the delay of the appropriate diagnosis and treatment for patients with ST elevation myocardial infarction (STEMI). 

ECG home monitoring studies were also conducted for patients with chronic atrial fibrillation (AF), which can cause peripheral thromboembolism leading to stroke [15,16]. These studies also showed the effectiveness of telecardiology services. In summary, these clinical studies showed that post-hospital patients can undoubtedly benefit from daily and routinely 12-lead ECG measurement and data transmission from home to hospital, because on-duty physicians can receive the needed data, detect new cases of AF, order antiarrhythmic pharmacotherapy, and monitor the possible drug-induced pro-arrhythmic effects via the remote ECG monitoring. 

The SHL telecardiology project recruited post-hospital patients with chronic heart failures, such as valvular-, hypertensive- , and ischemic-induced heart diseases [17]. The patients were instructed to transmit their 12-lead ECG to a hospital station via a telephone line for physicians’ interpretation on a daily basis. This routine home ECG monitoring enabled clinicians to identify high risk patients who need immediately treatment and to screen out low risk patients who do not need emergency services. This study proved that with this telecardiology service, the re-hospitalization rates, the length and the expenses of hospital stay, and the mortality rate of the patients with chronic heart failures were reasonably reduced. This study concluded that daily home monitoring of ECG data should be practiced so that clinicians and care-givers can detect warning signs of arrhythmias or heart failure and determine the necessity of re-hospitalization. 

The EPI-MEDICS is another project on ECG monitoring, especially for the purpose of pre-hospital assessment [18]. In this project, 3-lead ECGs or the derived 12-lead ECGs were measured by a portable ECG acquisition device, a personal ECG monitor (PEM), in different settings (*i.e.*, at work, at home, or on the trip). The PEM was equipped with an arrhythmic event alarm and was also embedded with a modem, which transmitted the measured ECG files and patients’ clinical history to a nearby hospital station via a wireless network so that cardiologists can make the diagnosis via mobile phones. This project was evaluated by physicians, cardiologists, and patients. The results showed the great advantage of pervasive telecardiology service, which facilitated pre-hospital diagnosis for the patients with acute coronary syndromes. 

To sum up, it is vital to develop and improve home-based monitoring services; however, it may not be easy to train patients to put on the electrodes and to transmit the data to the medical professionals. To solve this problem, it may be necessary to train family members or home-visiting assistants to operate the instruments and deliver the data to the remote medical professionals.

### 2.3. In-Hospital 12-Lead ECG and Imaging Teleconsultation

In 2009, a model of 12-lead ECG teleconsultation triage was developed in the Emergency Department (ED) of the Taoyuan Armed Forces General Hospital in Taiwan [19]. In this study, clinically-used 12-lead ECG devices, such as the HP 1770A and Philips Pagewriter series, were given additional functions to transmit ED patients’ ECGs to the mobile phones of remote cardiologists for immediate interpretation. That is, off-site senior cardiologists can receive and read patients’ 12-lead ECGs via their smart phones if an emergency teleconsultation call is requested by the on-site ED physicians. Additionally, off-site cardiologists can access patients’ historical ECG reports and lab reports stored in hospital information system (HIS) via smart phones. With the ubiquitous access of ECGs and relevant medical files in HIS, off-site cardiologists can make timely and appropriate medical decisions and guide the on-site ED physicians to prepare for the necessary treatment before they return to the hospital. Different from the traditional ECG telephone teleconsultation where the on-site physician describes the ECG waveform to the off-site cardiologist via phone conversation, this 12-lead ECG teleconsultation technology greatly enhances the efficacy of emergency medical services. 

In 2013, another 12-lead ECG teleconsultation triage model was developed in the ED of Foggia University Hospital in Italy [20]. In this study, 12-lead ECGs of the ED patients suffering with chest pain were transmitted, in real time, to the central telecardiology hub via a Cardio Vox-P12 ECG recorder device. Consequently, a remote senior cardiologist can receive the transmitted ECGs from a fax and interpret the data. If acute myocardial infarction (AMI) is confirmed, the catheterization lab is promptly alerted and the patient will be given percutaneous coronary intervention (PCI) within 30 min. Several other clinical trials indicated that timely and accurate 12-lead ECG interpretation did help on-site paramedics conduct necessary PCI in 90 minutes, which critically reduced the mortality rate of patients with AMI [21,22].

**Figure 2:**
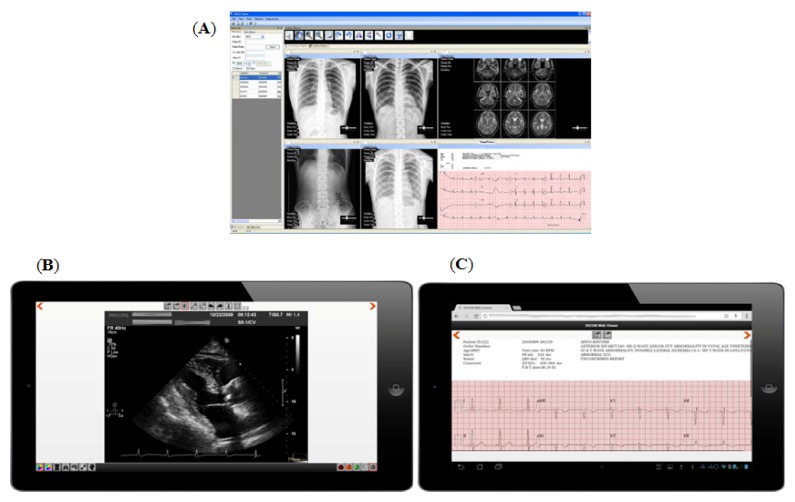
(**A**) Shows the manipulation of 12-lead ECG, X-ray chest, and MRI on a clinically-used PACS. (**B**) Shows a captured ultrasound image in an ECHO study using PACS via 3G telecommunication on a tablet. (**C**) Shows a display of 12-lead ECG through the access of PACS via 3G telecommunication on a tablet.

Chest X-rays are another key diagnostic tool for cardiologists to differentiate non-specific chest pain and coronary disease-induced chest pain. To make the correct diagnosis for patients with chest pains at ED, off-site cardiologists need more reference data, including chest x-rays and 12-lead ECG data. In Hsieh and colleagues’ study in 2010, ED patients’ chest x-rays and 12-lead ECGs were delivered to the off-site cardiologists’ mobile phones [23]. With this ED 12-lead ECG teleconsultation model, heterogeneous formats, including HP SCP-ECG and Philips XML-ECG, were integrated into a unified format based on the open standard, Digital Imaging Communication System on Medicine (DICOM) [23,24]. In this way, 12-lead ECGs can be stored in Picture Archiving and Communication Systems (PACS), which are an archiving system for the storage of the medical images generated from imaging based modalities in hospitals [23,25]. When an emergency teleconsultation is needed, off-site cardiologists will be contacted and ubiquitously access patients’ present and historic 12-lead ECG, x-ray chest, and other relevant images in PACS to make the correct diagnosis and recommendation. As shown in Figure 2A, 12-lead ECG and x-ray chest were stored in PACS and can be displayed by a DICOM viewer. In Figure 2B,C, an application (APP) called mobile PACS developed by Hsieh’s lab can gain access to PACS via wireless network so to visualize patients’ ECHO and 12-lead ECG in tablets. In addition to the telecardiology consultation purpose, the integration of 12-lead ECG and medical images in PACS can enhance the efficiency of patient data management, as indicated by the subsequent study in 2012 [26].

### 2.4. The Teleconsultation of Intensive Care Unit (ICU) and Coronary Care Unit (CCU)

ICU patients are vulnerable to sudden death, which makes ICU patient care critical. Several clinical trials have indicated that tele-ICU technology can effectively reduce the mortality rate and the length of hospitalization [27,28]. Tele-ICU is carried out by experienced nurses, and cardiologists, who are responsible for the 24-hour continuous remote monitoring of patients’ vital signs, including 3-lead ECG or derived 12-lead ECGs, blood pressure waveforms, oxygen saturation (SpO2), carboxyhemoglobin concentration (SpCO) in blood, respiration waveforms, and body temperature. This tele-ICU technology works like a second set of eyes of bedside paramedics [29]. To perform remote monitoring, ICU patients’ vital signs are transmitted to a remote tele-ICU commander center in real time. Another important feature of this tele-ICU technology is the real-time tele-communication and tele-consultation among the bedside paramedics, off-site professionals, and ICU patients via surveillance and the 24-hour alert system. To increase the efficacy of tele-ICU and bedside patient care, the tele-ICU team are provided with the remote access to the PACS, HIS, and nurses’ notes. 

CCU patients are at risk for sustained or nonsustained ventricular tachycardia (VT), myocardial ischemia or myocardial infarction, AF, atrioventricular block, or pacemaker malfunction, which may easily cause sudden death. CCU patients often experience the aforementioned complications and die when bedside paramedics treat other patients and fail to note patients’ emergency alarms. Because of the shortage of nurses and medical specialists in CCU, tele-CCU services are in great demand where remote cardiologists and experienced nurses can help bedside clinicians to monitor patients’ conditions via telecommunication [30]. With the tele-CCU technology, patients’ vital signs measured by the bedside monitor are transmitted to a central database, and this database can be accessed by the tele-CCU team via internet. In 2009, the feasibility of a tele-CCU model at Tampere University Hospital in Finland was proved through technical and clinical evaluation [30]. In this study, the beside monitor, Philips IntelliVue, can export vital signs to a database where the clinical data are remotely accessed and monitored by a group of experienced cardiologists in real time. The remote cardiologists observe ECG waveforms and other vital signs and provide bedside clinicians with real-time interactive tele-consultation and medical order for treatment. 

The remote monitoring of vital signs of CCU patients was further developed into ubiquitous monitoring by several researchers in Japan [31]. With the monitoring, CCU patients’ vital signs measured by a bedside Fukuda Denshi Model DS-7100 monitor were transmitted to the remote mobile phones via a 3G wireless network. The off-site cardiologists who monitor patients’ vital signs gave the necessary medical treatment order to the bedside nurses and physicians.

### 2.5. Pre-Hospital 12-Lead ECG Diagnosis

In 2008, American College of Cardiology and American Heart Association recommended the application of 12-lead ECG onto pre-hospital diagnosis [32]. For example, 12-lead ECGs of patients with chest pain are delivered from a moving ambulance to the hospital station so that timely and appropriate treatment for patients with STEMI can be offered. Therefore, pre-hospital 12-lead ECG diagnosis is useful to dispose patients with chest pain. 

**Figure 3:**
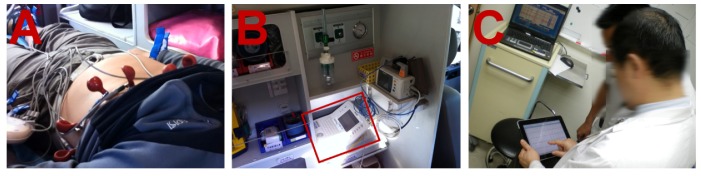
The demonstration of pre-hospital 12-Lead ECG diagnosis.

Grim and his associates in 1987 first demonstrated the transmission of 12-lead ECG from a moving ambulance to a hospital station [33]. In Grim *et al.*’s study, a portable 12-lead ECG device, Marquette’s ECG, linked with a modem and a cordless phone, was installed on an ambulance. The digitalized ECG waveforms were sent back to a receiving ED station prior to the ambulance’s arrival at the hospital. Pavlopoulos and his associates in 1998 successfully developed a mobile telemedicine system installed on an ambulance for the application of pre-hospital diagnosis [34]. In their study, 3-lead ECGs, heart rate, SpO, SpCO and still images of patients were transmitted via a wireless network and a modem from a moving ambulance. These data were delivered to the hospital station in real time. In the following years, a number of clinical trials demonstrated successful transmission of 12-lead ECG with the use of defibrillators [35,36,37]. In these clinical trials, 12-lead ECGs were transmitted by the defibrillators which were optionally embedded with the 12-lead ECG module, such as Physiol-Control’s LifePAK series, linked to a modem via a wireless network. ECGs were first sent to the hospital station and then faxed to a cardiologist for pre-hospitalization ECG interpretation and diagnosis. In 2010, Hsieh and his associates developed a simple technique to transform a hospital routinely-used 12-lead ECG device, the Philips Pagewriter, into a portable ECG device [38]. Hsieh and his associates enabled this ECG device to transmit 12-lead ECGs via a 3G wireless network from a moving ambulance to a hospital station or to the off-site cardiologists’ mobile phones. This newly developed portable ECG device and the pre-hospital ECG diagnosis technology were used by the Department of Cardiology at the Far Eastern Memorial Hospital in Taiwan, as shown in Figure 3. In panel A of Figure 3, a patient is attached with 12-lead ECG electrodes on an ambulance. In panel B of Figure 3, a clinically-used 12-lead ECG device, Philips Pagewriter, which serves to transmit ECG XML file via 3G wireless network, was installed on an ambulance to transmit 12-lead ECGs. In panel C of Figure 3, a cardiologist can receive an ECG report transmitted from a moving ambulance via an iPad tablet. Several clinical trials have validated the advantages of pre-hospital 12-lead ECG diagnosis for patients suffering from STEMI, including the reduction of door-to-balloon (D2B) time, faster time for reperfusion, and timely thrombolytic administration [39,40,41]. 

## 3. The Development of Tele-ECHO

Traditionally, clinically-used ECHO was equipped with an export of S-video with which each examination was recorded and stored on a videotape for the subsequent teleconsultation called store-and–forward teleconsultation, or the video was digitalized, compressed, and sent to a remote cardiologist via network as real-time teleconsultation [42,43,44]. The modern ECHO modalities generate a sequence of DICOM based images and export the digital video files for store-and-forward interpretation or real-time examination. As compared to the traditional ECHO system, the cardiologists can benefit more greatly from the DICOM-based ECHO, the advantages of which include rapid data retrieval via PACS, convenient comparison with previous examination, and shared access to the data among cardiologists [42]. 

Finlay *et al*. published the first use of real-time tele-ECHO research via video transmission in the late 1980s [45]. In 1996, Trippi and his associates developed a model of store-and-forward ECHO teleconsultation called ED ECHO teleconsultation, where ED patients’ still ECHO images were transmitted to a laptop computer of a remote cardiologist who examined the ventricular functions and valvular diseases via the traditional telephone line linked to a computer modem [46]. In subsequent years, real-time ultrasound imaging transmission technology was developed; with which cardiologists can remotely guide the sonographer to manipulate the ultrasound probe to acquire useful information via videoconference. Cardiologists then have the acquired images transmitted in real-time via ISDN with low 384 kb/s bandwidth [47]. Recent studies indicated that both the real-time tele-ECHO and the store-and-forward tele-ECHO are especially useful for the rural areas where few pediatric cardiologists are available [48,49,50,51]. Tele-ECHO can avoid the unnecessary and expensive patient transportation, result in timely medical intervention, lower the mortality rate, and greatly reduce medical costs [48,49,50,51]. As indicated by recent studies, echocardiography has developed to mobile phone-based tele-ECHO [52,53]. Several mobile phone echocardiography modalities, such as GE Vscan and MobiSante MobiUS, enable sonographers to conduct ultrasound examination with real-time image transmission via Wi-Fi or 3G mobile phones, while the images are stored in a PACS. The quality of transmitted images recorded by mobile phones has been proven to be acceptable [54,55]. The mobile phone-based tele-ECHO will expand the applications of tele-ECHO into pre-hospital ECHO and home monitoring ECHO, while a few problems are yet to be overcome, including the training of emergency medical technician (EMT) and adult patients, who need to manipulate the ultrasound device and acquire useful images for cardiologists’ interpretation. This is important because a successful tele-ECHO examination depends on the ability of a sonographer to acquire proper images, with which cardiologists can evaluate the size of heart chambers, valvular anatomy, and intra-cardiac hemodynamics. For real-time tele-ECHO, audio-visual communication system may be an important tool because cardiologists can use it to direct sonographers to find the correct examination location and reduce the unnecessary repetitive but ineffective examination.

## 4. New Framework of Telecardiology

**Figure 4:**
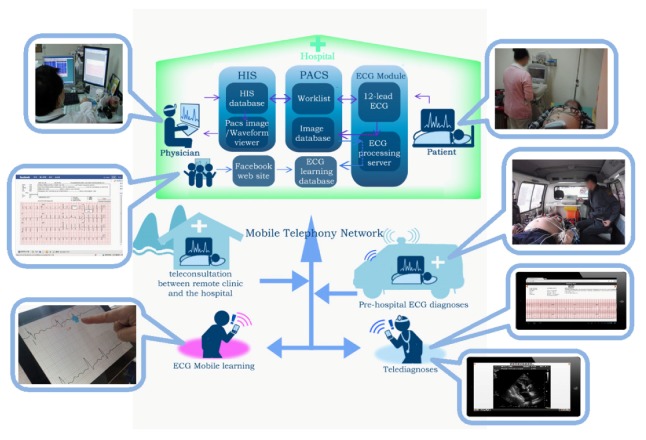
Mobile computing based telecardiology.

Modern mobile computing technology can advance telecardiology with pervasive ECG, ECHO, and relevant medical image applications as described in Section 2 and Section 3. As shown in Figure 4, mobile computing based telecardiology services, including 12-lead ECG and imaging tele-diagnosis through the access of PACS, pre-hospital ECG diagnosis, 12-lead ECG e-learning, on-line ECG discussion through Facebook social network, and teleconsultation between a clinic and a hospital, have been developed by Hsieh’s lab since 2009 [8,19,23,38]. However, the delivery of telecardiology services to an experienced cardiologist for timely and effective interpretation remains a great challenge. Most telemedicine services are offered only within a hospital, between a clinic and a specific hospital, or between under-resourced clinics and a metropolitan hospital, which are not wide enough services. Moreover, the time of the services is often restricted by the availability of experienced cardiologists. To overcome these limitations, a common telecardiology platform with shared access nationwide or even worldwide is in great demand. If different clinics, hospitals, medical centers around the world can team up and establish a databank including ECG, ECHO, and relevant images, and if there are sufficient senior cardiologists from different countries who can take part in the telecardiology consultation teamwork, it will be possible to carry out ubiquitous national and international emergency teleconsultation among rural clinics, hospitals, medical centers, professionals and at-home patients, or even moving ambulances. Meanwhile, hospitals and medical centers will face a serious problem with the storage of rapidly increasing medical data. For example, a critical CCU patient can generate 1 GB data or more per day including continuous 3-lead ECG or 12-lead ECG and other relevant vital signs. For example, the size of a 640 × 480 pixel RGB frame in the DICOM format is about 1 MB, and a 15-min ECHO study with 30 frames per second would occupy over 25 GB [42,56]. To solve the storage problems, hospitals administrators need to adopt an outsourcing strategy of storing ECG and medical images. An example of this outsourcing strategy is the concept of storage-as-a-service cloud computing, which provides hospitals with a big data storage capacity based on their specific demands at a low cost. The strategy of storage-as-a-service cloud computing not only resolves the storage problems but also facilitates big data analysis [57,58,59]. 

### 4.1. Cloud Computing Can Benefit Telecardiology and Large-Scale Medical Data Analysis

Cloud computing is a relatively new model of delivering computing resources, which consists of processing, memory, storage, and network. These resources are virtualized, configurable, and accessible by users with Web via the internet [60]. In cloud computing, user-developed applications are run on a virtual machine (VM), which has the functions of a real computer. The functions of VM are continuously monitored by the cloud to ensure the applications are always available and accessible. While being virtualized for users in the cloud, these computing resources can be adjusted based on users’ demands with high scalability. It should be noted that cloud computing users are given immediate access to computing resources as utility computing at a low cost. The high availability, accessibility, and scalability are the key characteristics of cloud computing technology. Importantly, most cloud computing providers can routinely back up data and store the duplicated data at different datacenters of the cloud providers. In this way, data can be recovered in case of machinery damages or malfunctioning. To safeguard users’ data, cloud providers offer solid and advanced firewall to filter out break-in activities of unauthorized users. From the perspective of hospital management, these cloud services have multiple benefits as mentioned above, which are superior to the traditional methods of clinical data management and storage. From the perspective of research development, scientists and clinicians from all over the world can take advantages of these cloud services as they can perform computing-intensive data analysis and share big data in the cloud. 

Since 2012, a 12-lead ECG telemedicine service in the cloud, including ECG signal processing, ECG data management, and teleconsultation, has been developed and proved to be feasible and useful by Hsieh and his associates [8]. In this study, the traditional ECG teleconsultation was expanded to facilitate the collaboration of two hospitals. This 12-lead ECG telemedicine service in the cloud offered convenient and affordable clinical data management, such as ECG interoperability when a patient was transferred from one hospital to the other, as well as effective ECG teleconsultation with prompt ECG delivery from the hospital to the mobile phones of off-site experienced cardiologists. 

The concept, or the term, ‘big data’ refers to an analysis technique associated with data mining. For example, the analysis is not only based on short-term current data, but also on long-term historical data and other relevant data. As compared with traditional data analysis, the big data computing technique requires large-scale data capacity. With respect of large scale data research, Chia and his associates employed big data computing to generate a predictor of the mortality risk for patients with acute coronary syndromes in 2011 [61]. This predictor was developed through data mining and machine learning, based on 24-hour continuous ECG readings over 4,000 patients’ trials. In each trial, 24-hour ECG readings were collected in a two-year period. This big data based predictor can predict over 50% deaths with fewer false positives as compared with the traditional ECG analysis, conducted based on a smaller segment of ECG signals. Another example is the use of cloud computing in the research of radiation therapy. Radiation therapy and image analysis produce a large amount of images files. To conduct large scale data research, scientists and clinicians need heavy calculation with data-intensive and compute-intensive computing resources. Keyes and his associates in 2010 successfully demonstrated a medical image analysis technique using Monte Carlo simulation with compute-intensive resources in the cloud [62], which takes the advantages of convenient access to and of the cost-effective storage of the computing resources.

### 4.2. A Cloud-PACS Extended from Local to Global

Cloud-PACS, which implements PACS on the infrastructure of cloud computing and serves as a public accessed software-as-a-service (SaaS) via the internet, is a critical technology for the revolution of telecardiology [58,63,64,65]. SaaS is one of the service models of cloud computing, which allows users-developed applications running on a VM of cloud vendors’ infrastructures and can be accessed by general public via internet through web browsers. With PACS, clinicians can index the images of the same or similar diagnosis and share the image files across hospitals. The future direction is the collaborative development of telecardiology service via the platform–as-a-service (PaaS) with Cloud-PACS by hospitals and academic institutes, where cloud computing providers offer hospitals with the computing environment for application development, networking, and storage without purchasing hardware and software. It should be noted that these telecardiology services are cost-effective. 

**Figure 5:**
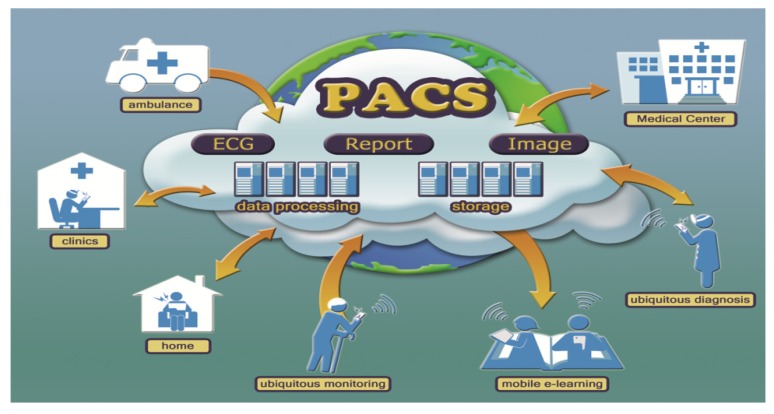
A global ECG and image cloud service for tele-consultation, research, and education.

Recent studies have indicated that there are several open source PACS implemented successfully in VM and the infrastructures of cloud computing, which can replace the traditional expensive PACS [58,66,67]. If an under-resourced clinic is unable to purchase the traditional PACS or to continuously collaborate with well-resourced hospitals for telecardiology services, it can take the advantage of Cloud-PACS services through SaaS on the basis of pay-as-you-go without long term commitment. As Figure 5 shows, Cloud-PACS can easily facilitate the interoperability of ECGs and images in a unified data format, DICOM, and allow rural clinics and metropolitan hospitals to use the service on the basis of pay-per-use. Importantly, this cloud-PACS service enables hospitals to store a large amount of images and ECGs offsite at a low cost. In addition, this technology can serve for both research and education purposes if each ECG or image interpretation is confirmed by cardiologists with an explicit report. Finally, this service can be easily extended from a local to a global level, with which clinics, hospitals, and medical centers around the world will share ECG, ECHO, and image files for the collaboration of pre-hospital, in-hospital, and post-hospital diagnoses and consultation.

## 5. The Feasibility of the Cloud Telecardiology Service

With the use of cloud computing and mobile computing, revolutionary ubiquitous telecardiology services can be expected in the near future. To date, there are several technical difficulties to overcome: (1) data confidentiality and information security in the cloud, (2) network latency, reliability, and accessibility, and (3) the short battery life, small storage capacity, and limited processing ability of mobile devices. In the following sections, we will discuss the feasibility of realizing Cloud-PACS and global telecardiology service platform, as well as the potential solutions to the problems. 

### 5.1. The Security of Data Transmission via Public Network

In the design of telecardiology services, researchers must ensure the transmission of patients’ medical data over public networks is secure. To date, there is no cryptography algorithm which guarantees 100% security of transmitting data via the internet. However, many algorithms can reduce the risks of data sniffers due to malicious internet attacks. A common method to retrieve medical data securely is called certificate-based secure data transmission, often used in web banking in the financial industry. The digital certificate is a digital document used to prove the authorized identity, which is embedded with a user’s private key and a public key for data encryption and decryption [68]. To perform certificate-based data transmission, a trusted Certificate Authority (CA), originating from telecardiology service providers will issue the digital certificate to authorized users to access the services via the internet. When a cardiologist accesses the telecardiology service via the internet, the service will authenticate the digital certificate, which can be implemented on the mobile phone of the cardiologist. Next, this service allows the ECG or image to be sent and received via Secure Socket Layer (SSL) over TCP/IP where the data packets are encrypted with a secret key. Another method for secure data transmission is called virtual private network (VPN). VPN allows users to connect to the service via a public network as if this connection were point-to-point via a private network, which consequently prevents data attack. To date, the combination of SSL and VPN are found to be effective for data security in the practice of telemedicine [69].

### 5.2. Privacy Protection in the Cloud

Obfuscation is a recently developed technique which directly processes the encrypted data and restore correct results by de-obfuscation [70,71]. Obfuscation can also selectively encrypt partial data contents and leave others unencrypted. For example, a DICOM-ECG or an image file can be encrypted with sensitive attributes, such as patients’ demographic information before it is sent to the cloud. The remote cardiologists can retrieve this ECG or image through privacy manager software, which is the key to obfuscation and de-obfuscation and is only known by the user, the authorized remote cardiologist whose mobile phone is implemented with the certificate. Because data are in the encrypted form in the cloud and the key to decryption is kept on the client side, data privacy is ensured through obfuscation. 

### 5.3. Data Confidentiality in the Cloud

In Hsieh *et al*.’s study, a data validation mechanism in the cloud database was developed to ensure patients’ ECG data integrity [8]. Data retrieval from this database was examined through cryptography to ensure data integrity. Data exchange between VMs was also safeguarded by SSL to ensure data integrity while data was processed in VMs. 

### 5.4. Cloudlet Can Improve Network Latency

Cloud-PACS services are often far from the location of users of mobile devices. As a result, images transferred from Cloud-PACS to the mobile devices via wide area networks (WANs) may cause image latency. To reduce image transmission latency via WANs, Satyanarayanan proposed a framework of cloudlets, which can be delivered as hardware and software with processor and memory functions, and can be embedded at various Wi-Fi access points [72]. In this framework, the Wi-Fi access point is connected to cloud providers via a high speed wire connection and therefore, it provides users with a higher speed Wi-Fi bandwidth than possible with a 3G mobile network. If this concept is realized, an off-site cardiologist can easily find a nearby cloudlet, which preloads the images from the remote data center of cloud providers, and use the cloudlet to perform image teleconsultation with less processing delay and transmission latency. 

### 5.5. Next Generation Network/IP Multimedia Subsystem (NGN/IMS) Can Enhance Network Reliability and Accessibility

In Rikitake’s study, the real-time transfer of ECG and other vital signs through NGN/IMS was conducted successfully [73]. NGN is a service-oriented network delivering data in various forms including audio and video files based on IP. The delivering process is controlled by transfer-related functions developed in NGN, which is more secure and reliable than the traditional network architecture [74]. NGN has the ability to enhance the security of data delivery through the use of authentication, authorization, and accounting (AAA), and it can also ensure sufficient bandwidth through the quality of service (QoS). These functions thus decrease the transmission latency and reduce data loss [73,74]. IMS is a network access technology of data interoperability across various networks, such as wireless network, the traditional telephone line, and WAN [74]. Not only may NGN/IMS be a simple and effective solution to the quality and security of ECG and images transmission over public networks, but it expands the networks from the traditional telephone line to various wire- and wireless networks, which realizes the mobility of healthcare application. 

### 5.6. The Image Compression Technology Can Speed up Image Transmission

JPEG 2000 and H.264 are recently developed image compression and video compression technologies [75,76]. In Kim and his associates’ study in 2011, a telemedicine application via mobile phones where patients’ images in DICOM format were converted into JPEG 2000 with the compression ratio of 1:10 was demonstrated, and the transmitted files were successfully sent to a remote radiologist for teleconsultation [77]. In Pederson *et al.*’s study in 2009, echocardiography experts confirmed the diagnostic quality of H.264-encoded ECHO videos as compared to the original ECHO videos [78]. These studies showed that with JPEG 2000 or H.264, a remote expert can receive images or ECHO more promptly, and the quality of the compression ratio did not differ significantly as compared with the original DICOM. In summary, JPEG 2000 and H.264 may be applied in ED emergency where image teleconsultation is needed. This technology is especially beneficial if the bandwidth is not wide enough.

### 5.7. Cyber Foraging Can Improve Computing Efficiency of Mobile Devices

When receiving and interpreting medical images on the mobile phone, the clinician needs to use the functions of interactive zooming, rotation, contrast adjustment, and measurement of area and volume, but it is a challenge to manipulate medical images without delay on smart phones. Recently, several researchers have proposed the concept of cyber foraging, which enables the mobile device to off load the compute-intensive tasks to a cloud, which later transfers back the processed results to the mobile device [79,80]. Obviously, mobile devices in telemedicine can take advantage of this technology. The benefits include increasing computing efficiency and battery life savings. The technology of server-side image rendering which enables images to be downloaded and processed in the remote data center of a cloud provider is similar to the concept of cyber foraging. This technology also allows users to visualize the processed images via a web browser. With the use of server-side image rendering, the performance of medical image transmission and tele-diagnosis requires only a minimum of the stable 4 Mb/s bandwidth [64].

### 5.8. DICOM and Cloud-PACS Can Facilitate the Interoperability of ECG and Medical Images

Interoperability is conceptualized and categorized as three levels, including data interoperability, system interoperability, and device interoperability. First, data interoperability refers to the ability of data sharing and re-use between hospitals. Unlike DICOM medical images with unified and open standard data format, ECGs have heterogeneous data formats [81]. These heterogeneous formats hinder the development of ECG interoperability. To achieve ECG data interoperability, several researchers have decoded SCP-ECG to extract ECG raw data and converted SCP-ECG to DICOM-ECG or FDA defined XML through the help of OPEN-ECG, which is a non-profit organization founded by European scientists [82,83,84]. Hsieh and his associates also decoded ECG files generated from common ECG devices, such as Philips Pagewriter series and HP 1770 series, to develop an emergency ECG teleconsultation technology [9,23]. We hope that ECG data interoperability can be unified with an open standard, which will allow clinicians and scientists to share the access, storage, and management of clinical files. Before this goal is achieved, much collaboration among hospitals, academic institutes, and associates in cardiology industry is critically required. 

Second, system interoperability refers to the ability to exchange, store, and manage ECG or image files among various information systems across hospitals. For example, inexpensive Cloud-PACS should be promoted with great efforts. If most hospitals adopt Cloud-PACS to manage medical images, system interoperability will be realized. 

Third, device interoperability refers to the ability to manipulate ECG and images on various mobile devices, such as Android-, IOS-, and Windows-based mobile phones. Recently developed HTML5 technology enables IT to develop an APP with HTML5 language and to perform the APP on various platforms via a web browser [85,86]. A telecardiology service with cloud computing and HTML5 technology can realize device interoperability. 

### 5.9. Diagnostic ECG and Image Reports Can Facilitate the Development of ECG and Image Research and Education

A cloud-based telecardiology platform can not only facilitate ubiquitous tele-consultation regionally or internationally, but it also promotes better research and education opportunities. This platform should consist of raw images, ECG signals, and medical reports where experienced cardiologists denote the abnormalities of images and files and store the data, and researchers and clinicians can retrieve these files for further analysis or educational purposes. When a cloud-based telecardiology platform is established, the data of images, ECG, and medical reports should also be coded anonymously to protect patients’ privacy. However, most diagnostic reports differ greatly in writing styles, length, and structure. These reports are usually filed in different information systems where images and ECG are stored separately. Consequently, it is difficult for clinicians or researchers to compare the reports with the matching images and ECGs. If a PACS service can include diagnostic reports, the advancement of research and education in telecardiology will come true. 

The National Electrical Manufacturers Association (NEMA) has defined various structured reporting (SR) specifications to improve the documentation of diagnostic waveforms and images in clinical domains, such as echocardiography SR and ECG SR [87,88]. In data format, SR is similar to DICOM-based images, which is composed of information elements with numerical codes and organized as a tree-like structure, and thus SR can be completed, stored, and interoperated through PACS [89,90]. SR is a NEMA-proposed model for image or waveform interpretation and documentation procedures, which guides clinicians to conduct an explicit diagnosis and document reports by checking and answering each information element (*i.e.*, codes) defined in SR. With SR, clinicians denote their findings with text documents and the specific codes representing a particular region of images and waveforms. In this way, the diagnostic reports can be easily referenced and compared with other reports, as well as ECG or image files of the same or different patient with the same reference code. Research showed the great benefits of using SR in clinical practice, which makes medical reports more explicitly written, easily referenced, and conveniently stored and managed with the matching ECG and image files. Additionally, the codes in SR can be easily indexed and searched by computer algorithms. As a result, data mining of diagnostic images and ECG, or even big data research, can be conducted. If a cloud platform is developed in this way, researchers can perform large scale clinical research ubiquitously, and young clinicians can review and learn from the reports and files through e-learning. 

As required by the community of biocuration for exponential growth of gene data, a bioinformatics study developed a unified structure to document genome findings, which extracted and tagged researchers’ finding [91]. This is an example of a unified and explicit structure used to document the findings in gene and protein research, which established a sound knowledge database for advanced big data research. Despite of several studies’ validation of the importance of using SR when clinicians conduct diagnosis of clinical images, many clinicians may have problems adopting SR in their routine diagnostic report documentation [92,93]. First, it may be time-consuming for a cardiologist to complete an ECHO SR or ECG SR. Second, cardiologists may find it troublesome to change their traditional documentation practice to the new SR requirements.

## 6. Conclusions

There is no doubt that mobile teleconsultation and cloud computing are two central components of modern telecardiology. In our consideration, PACS should also be applied to cloud computing because PACS facilitates data interoperability of ECGs, image files, and diagnostic reports. With data interoperability, a clinical consultation, research, and educational platform can be established where regional and international clinicians, researchers, educators, and students can upload or download clinical data including ECGs, image files, and medical reports to review and discuss pathological signs and diagnostic results. If documented, formatted, and stored with the same standard, clinical data will be easier to reference and compare. If there are enough senior cardiologists from around the world who take part in the consultation team on the platform, 24-hour teleconsultation will be possible. With the accumulation of data, big data research will be doable. 

Cloud computing has the benefits of highly efficient computing performance, cost-effectiveness, and sufficient storage for data delivery and management. In cardiology, cloud computing technology and mobile teleconsultation should be combined because mobile teleconsultation requires high speed data delivery and a big data center where data can be delivered, stored, retrieved, and managed securely. As patients in cardiology may need hospitalization or re-hospitalization and are often vulnerable to sudden death, it is critical for medical professionals to provide pre-hospital, in-hospital, and post-hospital patients with timely and appropriate diagnosis and treatment. However, timely medical services are not always available not only in rural areas but also in metropolitan hospitals. In addition, post-hospital patients, ambulance paramedics, as well as on-site medical professions including ED physicians and bed-side nurses, often need to seek emergency consultation from experienced cardiologists who are off-site. Consequently, tele-consultation, especially mobile tele-consultation, seems to be a good solution to emergency medical situations. Meanwhile, cloud computing with PACS can facilitate data interoperability, and therefore, to enable timely delivery of all needed data for emergency teleconsultation between professionals, between patients and clinicians, and between hospitals. Before the aforementioned goals are reached, the following tasks need to be completed: the first task is to ensure data security in the cloud. The second task is to deploy more secure and reliable public networks. The third task is to increase the motivation for clinicians and manufactures to collaborate and adopt the new ECG devices or imaging modalities that are interoperable, which will then promote the system interoperability among hospitals. If these issues are resolved, the ubiquitous platform services will be easy to use, inexpensive, and beneficial to clinicians, researchers, and students. Most importantly, these services will increase global collaboration to allow cardiologists to better communicate with non-specialists within their regions, or from various regions and nations, and also to facilitate the work of charitable organization such as Swinfen to provide expertise to developing countries [94]. In this way, the clinical practice and scientific research in cardiology will be advanced more rapidly worldwide.
